# A *nadA* Mutation Confers Nicotinic Acid Auxotrophy in Pro-carcinogenic Intestinal *Escherichia coli* NC101

**DOI:** 10.3389/fmicb.2021.670005

**Published:** 2021-06-02

**Authors:** Lacey R. Lopez, Cassandra J. Barlogio, Christopher A. Broberg, Jeremy Wang, Janelle C. Arthur

**Affiliations:** ^1^Department of Microbiology and Immunology, The University of North Carolina at Chapel Hill, Chapel Hill, NC, United States; ^2^Department of Genetics, The University of North Carolina at Chapel Hill, Chapel Hill, NC, United States; ^3^Center for Gastrointestinal Biology and Disease, The University of North Carolina at Chapel Hill, Chapel Hill, NC, United States; ^4^Lineberger Comprehensive Cancer Center, The University of North Carolina at Chapel Hill, Chapel Hill, NC, United States

**Keywords:** inflammatory bowel disease, adherent invasive *E. coli*, *Escherichia coli*, auxotrophy, nicotinic acid, *E. coli* NC101

## Abstract

Inflammatory bowel diseases (IBDs) and inflammation-associated colorectal cancer (CRC) are linked to blooms of adherent-invasive *Escherichia coli* (AIEC) in the intestinal microbiota. AIEC are functionally defined by their ability to adhere/invade epithelial cells and survive/replicate within macrophages. Changes in micronutrient availability can alter AIEC physiology and interactions with host cells. Thus, culturing AIEC for mechanistic investigations often involves precise nutrient formulation. We observed that the pro-inflammatory and pro-carcinogenic AIEC strain NC101 failed to grow in minimal media (MM). We hypothesized that NC101 was unable to synthesize a vital micronutrient normally found in the host gut. Through nutrient supplementation studies, we identified that NC101 is a nicotinic acid (NA) auxotroph. NA auxotrophy was not observed in the other non-toxigenic *E. coli* or AIEC strains we tested. Sequencing revealed NC101 has a missense mutation in *nadA*, a gene encoding quinolinate synthase A that is important for *de novo* nicotinamide adenine dinucleotide (NAD) biosynthesis. Correcting the identified *nadA* point mutation restored NC101 prototrophy without impacting AIEC function, including motility and AIEC-defining survival in macrophages. Our findings, along with the generation of a prototrophic NC101 strain, will greatly enhance the ability to perform *in vitro* functional studies that are needed for mechanistic investigations on the role of intestinal *E. coli* in digestive disease.

## Introduction

Inflammatory bowel diseases (IBDs), including Crohn’s disease and ulcerative colitis, are a major global health concern that affects over 3 million adults in the United States alone (Kaplan, 2015; Dahlhamer et al., 2016). IBD is a chronic and multifactorial disease that is driven by aberrant immune responses to commensal microbes, genetic susceptibility, and environmental factors (Khor et al., 2011). IBD patients experience painful, chronic, and relapsing intestinal inflammation that can lead to life-threatening complications, including intestinal fibrosis and colorectal cancer (CRC) (Rieder et al., 2007; Nadeem et al., 2019). Experimental models have demonstrated that IBD and CRC can be driven by the intestinal microbiota and that specific microbes, such as *Escherichia coli*, are associated with human disease (Schirmer et al., 2019; Lopez et al., 2021). IBD and CRC have no single etiology and no cure (Sartor, 2006; Goethel et al., 2018). Therefore, understanding the function of disease-associated gut microbes may uncover novel therapeutic options for intestinal diseases, such as IBD and CRC.

Intestinal microbes influence the onset and progression of IBD and CRC via metabolite production and modulation of mucosal immunity (Sartor, 2008; Dogan et al., 2014; Baümler and Sperandio, 2016; Hughes et al., 2017; Caruso et al., 2020). *E. coli* are common inhabitants of the intestinal microbiota (Tenaillon et al., 2010; Mirsepasi-Lauridsen et al., 2019). Strain level differences can alter the pro-inflammatory or pro-carcinogenic potential of *E. coli* partly through changes in small-molecule production (Arthur et al., 2012; Dogan et al., 2014; Baümler and Sperandio, 2016; Hughes et al., 2017; Ellermann et al., 2019). A pathovar of *E. coli*, termed adherent-invasive *E. coli* (AIEC), is enriched in the gut microbiota of human IBD and CRC patients (Martinez-Medina and Garcia-Gil, 2014). AIEC exacerbate experimental colitis and promote CRC in a variety of murine models (Carvalho et al., 2008, 2012; Arthur et al., 2012; Small et al., 2013; Martinez-Medina et al., 2014; Dejea et al., 2018; Ellermann et al., 2019). There is no genetic definition for AIEC (Nash et al., 2010; Dogan et al., 2014; Martinez-Medina and Garcia-Gil, 2014). Instead, AIEC are classically defined by their ability to adhere/invade epithelial cells and survive/replicate within macrophages (Darfeuille-Michaud et al., 2004; Martinez-Medina and Garcia-Gil, 2014). Environmental conditions, including nutrient availability and intestinal inflammation, can alter AIEC behavior and impact intestinal colonization and disease (Dogan et al., 2014; Ellermann et al., 2015, 2019, 2020; Delmas et al., 2019; Ormsby et al., 2020). Therefore, the ability to precisely manipulate AIEC growth conditions is essential for *in vitro* studies investigating AIEC behavior and production of pro-inflammatory and pro-carcinogenic molecules.

*Escherichia coli* NC101 is a well-known AIEC strain utilized by numerous investigators to study how intestinal *E. coli* adapt to and influence the host during IBD and CRC (Arthur et al., 2012; Tchaptchet et al., 2013; Dogan et al., 2014; Dejea et al., 2018; Ellermann et al., 2019; Gogokhia et al., 2019; Schmitz et al., 2019). NC101 was originally isolated from a specific pathogen-free wild-type (WT) mouse at the North Carolina State University (Kim et al., 2005). Colonizing WT mice with NC101 does not induce intestinal pathology, even during monoassociation studies using gnotobiotic animals (Ellermann et al., 2019). However, despite a lack of traditional toxins and virulence factors, NC101 induces antigen-driven intestinal inflammation in genetically susceptible IBD mouse models (e.g., interleukin 10-deficient mice) (Kim et al., 2005). Thus, NC101 is considered a pathobiont and a highly relevant model organism for defining how susceptible individuals may mount inappropriate immune responses to seemingly innocuous intestinal *E. coli*.

NC101 adapts to the inflamed intestinal milieu by modulating the expression of its gene repertoire (Patwa et al., 2011; Arthur et al., 2014). Nutrient availability alters AIEC physiology, persistence in the microbiota, and production of pro-inflammatory and pro-carcinogenic mediators (Dogan et al., 2014; Ellermann et al., 2015, 2019, 2020; Delmas et al., 2019; Ormsby et al., 2020). Monoassociation studies with gnotobiotic mice have led to the discovery of several AIEC-derived host-influencing molecules (i.e., specialized metabolites) that drive inflammation and tumorigenesis, including yersiniabactin and colibactin (Arthur et al., 2012; Martinez-Medina and Garcia-Gil, 2014; Ellermann et al., 2019). Like many specialized metabolites, yersiniabactin and colibactin are produced via biosynthetic gene clusters that can be activated by changes in micronutrient availability, notably iron (Perry and Fetherston, 2011; Tronnet et al., 2016). The nature of AIEC-derived specialized metabolites makes them difficult to isolate and study in functional assays. Therefore, the repertoire of AIEC-derived metabolites and their impact on the host has been largely unexplored.

Variations in micronutrient availability can impact the virulence and physiology of AIEC (Sharon et al., 2014; Ellermann et al., 2015, 2020). Therefore, culturing AIEC for mechanistic studies necessitates using a simplified base medium that allows for precise nutrient manipulation. During our studies, we observed that modified M9 minimal media (MM) does not sustain NC101 growth *in vitro.* We hypothesized that NC101 was an auxotroph. Through nutrient supplementation studies, we discovered that NC101 requires nicotinic acid (NA, niacin, vitamin B3) for growth. NA auxotrophy was not observed in other non-toxigenic laboratory *E. coli* strains (K12 or 25922), AIEC, or non-AIEC human intestinal strains (Baumgart et al., 2007). Genetic evaluation revealed that NC101 has a missense mutation in the nicotinamide adenine dinucleotide (NAD) biosynthesis gene (*nadA*) that encodes for quinolinate synthase A. Importantly, we generated a prototrophic NC101 revertant strain that eliminated *E. coli* micronutrient restraints. Correcting NC101 auxotrophy had negligible impact on NC101 function, including motility and AIEC-defining survival in macrophages.

NC101 micronutrient constraints have limited our ability to perform *in vitro* functional studies, which often require careful nutrient manipulation. Overall, our findings will enable precise nutrient manipulation for mechanistic studies on auxotrophic microbiota members, like AIEC, *Shigella* spp., or uropathogenic *E. coli* (Prunier et al., 2007b; Li et al., 2012; Di Martino et al., 2013; Bouvet et al., 2017). Importantly, our work will facilitate *in vitro* functional assays and small-molecule purification efforts with the pro-inflammatory and pro-carcinogenic AIEC strain NC101. Furthermore, these studies will broadly improve our understanding of the microbiota in intestinal diseases like IBD and CRC.

## Materials and Methods

### Bacterial Strains

Descriptions of *E. coli* strains used in this study are listed in Table 1. NC101 Δ*fliC* was generated using the λ-red recombinase method, as previously described (Datsenko and Wanner, 2000; Ellermann et al., 2019). Primers used for Δ*fliC* generation are listed in Table 2.

**TABLE 1:** *Escherichia coli* strains used in this study.

**Strain**	**Description of non-virulent *E. coli* strains**	**Isolated from:**	**Adherent-invasive *E. coli* (AIEC) Status**	**Reference or Source**
Wild-type (WT) NC101	Streptomycin-resistant (Str^*R*^) isolate of classical murine-derived AIEC	Laboratory *E. coli* isolate	AIEC	Kim et al., 2005, This study
LF82	Classical human-adapted clinical AIEC isolate	Crohn’s disease patient	AIEC	Darfeuille-Michaud et al., 1998
42ET-1	Clinical AIEC isolate	Non-IBD patient	AIEC	Baumgart et al., 2007
568-3	Clinical AIEC isolate	Crohn’s disease patient	AIEC	Baumgart et al., 2007
K12 (MG1655)	Model *E. coli* strain	Laboratory *E. coli* isolate	Non-AIEC	ATCC^®^ 700926^TM^
25922	Model *E. coli* strain	Patient	Non-AIEC	ATCC^®^ 25922
HM670	Clinical *E. coli* isolate	Crohn’s disease patient	Non-AIEC but has enhanced survival in macrophages	Martin et al., 2004
37RT-2	Clinical *E. coli* isolate	Non-IBD patient	Non-AIEC	Baumgart et al., 2007
532-9	Clinical *E. coli* isolate	Crohn’s disease patient	Non-AIEC	Baumgart et al., 2007
39ES-1	Clinical *E. coli* isolate	Crohn’s disease patient	Non-AIEC	Baumgart et al., 2007
Nicotinic acid revertant (NA_*derivative*_ or NA_*D*_) NC101	Spontaneous prototrophic revertant of WT NC101 (G263T mutation in *nadA*)	Laboratory *E. coli* isolate	ND but exhibits survival in macrophages	This study
NC101 Δ*fliC*	Non-motile flagellin mutant derived from WT NC101	Laboratory *E. coli* isolate	ND	This study

*IBD, inflammatory bowel disease; ND, not determined.*

**TABLE 2:** Primers used for strain construction.

**Primer**	**Sequence (5′–3′)**	**Reference**
Knockout_*fliC* forward	GGAAACCCAAAACGT AATCAACGACTTGCAAT ATAGGATAACGAA TCATGATT CCGGGGATCCG TCGACC	This study
Knockout_*fliC* reverse	GTCAGTCTCAGTTAATCAG GTTACGACGATTAACCCTG CAGCAGAGACAGTGTAG GCTGGAGCTGCTTCG	This study
*fliC* upstream	GACGATAACAGGGTTGACGG	This study
*fliC* downstream	ATTGCAATTCCCCTTGTAGG	This study

### Media Composition

#### M9 Minimal-Defined Media (MM)–5X M9 Salts

64 g Na_2_HPO_4_×7H_2_O, 15 g anhydrous KH_2_PO4, 2.5 g NaCl, and 5 g NH_4_Cl brought to 1 L in diH_2_O (Sambrook and Russell, 2006).

#### Complete MM

0.1 mM CaCl_2_, 1X M9 salts, 2 mM MgSO_4_, 0.4% glycerol, and 0.2% casamino acids (CAA, Sigma #2240) brought to 1 L in diH_2_O. Where indicated, the following were added at these final concentrations: NA (Sigma #N4126), 50 μg/L; L-aspartate (L-asp), 200 mg/L; nicotinamide (Nm), 50 μg/L; and NAD, 1 μg/L.

#### Vitamin Mix, 100X Stock

2 mg folic acid, 10 mg pyridoxine hydrochloride, 5 mg riboflavin, 2 mg biotin, 5 mg thiamine, 5 mg NA, 5 mg calcium pantothenate, 0.1 mg vitamin B12, 5 mg p-aminobenzoic acid, 5 mg thioctic acid, and 900 mg monopotassium phosphate brought to 1 L in diH_2_O and aliquoted into 10-ml stocks. One 10-ml stock was used per liter of media. Formulation is from ATCC and is based on Wolfe’s Vitamin solution (ATCC^®^ MD-VS^TM^).

### Overnight Cultures

Bacterial strains were preserved at -80°C and grown overnight at 37°C on Luria–Bertani (LB; Fisher Sc. #BP9722-2) agar plates. Isolated colonies were transferred to MM and grown overnight (>15 h) at 37°C with shaking at 220 rpm.

### Growth Assays

Overnight cultures were centrifuged and washed three times with 1X phosphate buffered saline (PBS) to remove any trace compounds contained in the culture. Cells were resuspended and normalized by optical density (OD_600_) in test media. Cultures were grown at 37°C with shaking at 220 rpm. For passaging assays, OD_600_ was recorded at the indicated time points (2, 4, 8, or 24 h). For growth curves, OD_600_ was recorded every 45 min for 6 h, and a final time point was recorded at 24 h.

### Spontaneous Prototrophic Revertant Generation

Wild-type NC101 was grown overnight in MM + NA, 5 ml of the culture was centrifuged, and the supernatant was discarded. The cell pellet was washed twice with 1X PBS and resuspended in 500 μl 1X PBS. A 100-μl spot was spread onto each of five MM agar plates without NA. Plates were incubated at 37°C and monitored for growth of revertant colonies (Li et al., 2012). Colonies were grown on MM without NA to confirm prototrophy, and isolates were preserved at −80°C. Whole-genome sequencing was performed to determine the location and nature of the mutation(s) leading to reversion. The revertant used in these studies was termed NC101 NA_*Derivative*_ (NA_*D*_).

### NC101 Genome Assembly

A complete NC101 genome was assembled from nanopore sequence using Minimap2 and Miniasm (Li, 2016). The assembly was circularized and polished four times with Racon (Vaser et al., 2017) followed by once with Medaka (Oxford Nanopore Technologies)^1^. Matched Illumina sequence data were used to polish the resulting assembly using FMLRC (Wang et al., 2018) with parameters “-k 21 -K 30 -m 3 -f 0.05 -B 10.” The final polished genome was rotated and linearized such that it starts at the origin of replication.

### Whole-Genome Sequencing

Three spontaneous prototrophic revertants, including NA_*D*_, were sent for whole-genome sequencing. Samples were sent to the Microbial Genome Sequencing Center (MiGS), formerly at the University of Pittsburgh, for genomic DNA extraction and Illumina 2×150 paired end sequencing on the NextSeq 550 platform. Sequencing reads were mapped to our closed NC101 genome using CLC Genomic Workbench7.5.1 with average coverage of 85× for JA0257, 62× for JA0265, and 75× for JA0266. Sequences for LF82 (NC_011993.1) and K12 (NC_000913.3) were obtained from the National Center for Biotechnology Information (NCBI), and all alignments were analyzed via Geneious Prime version 2020.1.2. Assembled sequences from this study were deposited in NCBI, and repository information is listed in Table 3.

**TABLE 3:** Repository information for published genomic sequences.

**Accession**	**Sample Name**	**Strain**	**Organism**	**TaxID**	**BioProject**	**Used in this study?**	**URL**
CP072787	JA0058	Original NC101 strain	*E. coli*	562	PRJNA678715	No	https://www.ncbi.nlm.nih.gov/nuccore/CP072787.1
CP070227	JA0072 (WT NC101)	Streptomycin-resistant (Str^*R*^) isolate of original NC101	*E. coli*	562	PRJNA678715	Yes	https://www.ncbi.nlm.nih.gov/nuccore/CP070227.1
CP072786	JA0257 (NA_*D*_ NC101)	Spontaneous prototrophic revertant of Str^*R*^ NC101 (G263T mutation in *nadA*)	*E. coli*	562	PRJNA678715	Yes	https://www.ncbi.nlm.nih.gov/nuccore/CP072786.1
CP072785	JA0265	Spontaneous prototrophic revertant of original NC101 (G263T mutation in *nadA*)	*E. coli*	562	PRJNA678715	No	https://www.ncbi.nlm.nih.gov/nuccore/CP072785.1
CP072784	JA0266	Spontaneous prototrophic revertant of original NC101 (T1014G mutation in *nadA*)	*E. coli*	562	PRJNA678715	No	https://www.ncbi.nlm.nih.gov/nuccore/CP072784.1

### Motility

Isolates were grown overnight, as described above. A 1-μl spot was used to inoculate the center of MM soft agar plates (MM + 0.25% agar) with NA. Plates were incubated at 37°C for 8 h, and the diameters of motility swarms were measured.

### Macrophage Survival Assays

Bacterial intramacrophage survival was measured using the standard gentamicin protection assay for AIEC bacteria (Darfeuille-Michaud et al., 2004; Ellermann et al., 2015). The J774A.1 murine macrophage-like cell line was used as a model and maintained according to ATCC standards in Dulbecco’s modified Eagle’s medium (DMEM) + 10% heat-inactivated fetal bovine serum (FBS) (DMEM, Gibco #11995-065). J774A.1 cells were seeded at 2 × 10^5^ cells/ml in 1 ml media into 24-well plates (Falcon #353047) and grown overnight. The next day, bacterial overnight cultures were subcultured in MM with and without NA for 3 h. Before infection, J774A.1 monolayers were washed twice with 1X PBS. Then, subcultured bacteria were added at a multiplicity of infection (MOI) = 10 in cell culture media with and without NA. Plates were spun at 180 × g for 5 min. Prepared bacterial cultures were serial diluted and plated on LB agar plates to validate infection dose.

After a 30-min incubation at 37°C with 5% CO_2_, infected cultures were washed twice with 1X PBS, and gentamicin-laden media was added (100 μg/ml gentamicin for 1 h time point and 20 μg/ml for 24 h in DMEM + 10% FBS with and without NA). At 1 and 24 h, cells were washed twice with 1X PBS, and 500 μl of 1% Triton X-100 in diH_2_O was added to each well for 5 min. Samples were mixed, serial diluted, and plated on LB agar plates to determine viable colony-forming units (CFUs). Percent intracellular bacteria = [(CFU/ml at 24 h)/(CFU/ml at 1 h] × 100.

### Statistics

Statistical analysis was performed using Prism version 9.0.0 (GraphPad software San Diego, CA, United States). A Welch’s *t*-test was used when two experimental groups were compared, and a one-way ANOVA with Dunnett’s T3 multiple comparisons test was used when three or more experimental groups were compared. Differences with a *p*-value less than 0.05 were considered significant. All experiments included at least three biological replicates with 1–2 technical replicates each, per time point.

### Data Availability

Assembled sequences from our whole-genome sequencing, above, were deposited in NCBI, and repository information is listed in Table 3.

## Results

### The Pro-carcinogenic Adherent-Invasive *E. coli* Strain NC101 Requires NA to Sustain Growth

**FIGURE 1:**
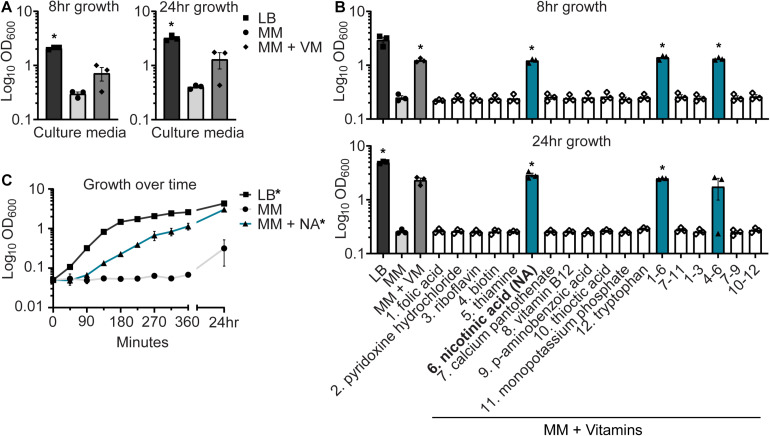
Nicotinic acid restores growth of *E. coli* NC101 in minimal media (MM). **(A)** Wild-type NC101 was grown in Luria–Bertani (LB) broth, MM, or MM + vitamin mix (VM). Growth was measured at 8 and 24 h by culture optical density, OD_600_. **(B)** NC101 was grown in LB, MM, MM + VM, or MM supplemented with individual or combinations of vitamins and tryptophan. OD_600_ was assessed at 8 and 24 h. **(C)** Growth curve of NC101 in LB, MM, or MM + NA. **(A,B)** Bars (*n* = 3) or **(C)** points (*n* = 4) depict mean ± SEM. Significance (*) is shown compared to NC101 growth in MM at **(A,B)** each time point or **(C)** 24 h and was determined at *p <* 0.05 using a one-way ANOVA with Dunnett’s T3 multiple comparisons test.

During *in vitro* studies to evaluate AIEC function in long-term culture (24+ h), we attempted to passage NC101 in modified M9 MM that includes glycerol and casamino acids. NC101 can successfully be subcultured from LB agar or broth, a rich medium, to MM (Ellermann et al., 2015, 2019). However, NC101 failed to grow when subcultured from MM to MM (Figures 1A–C). We hypothesized that NC101 was an auxotroph, unable to synthesize a key nutrient found in the murine gut. *Shigella* spp., a transient gut pathogen and close relative of *E. coli*, are generally NA (niacin, vitamin B3) auxotrophs (Ahmed et al., 1988; Prunier et al., 2007b; Di Martino et al., 2013; Bouvet et al., 2017). Thus, we specifically tested whether vitamin supplementation could restore NC101 growth in MM. Supplementing MM with a complex vitamin mix (VM) restored NC101 growth at 8 and 24 h (Figures 1A,B).

To identify which vitamins in the VM were essential for NC101 growth, we supplemented MM with individual or combinations of VM components and assessed NC101 growth. Tryptophan supplementation was also tested, as tryptophan metabolism can be influenced by host–microbe interactions in the gut (Agus et al., 2018). Only MM containing NA, alone or in combination, sustained NC101 growth in MM at 8 and 24 h (Figure 1B). Furthermore, NA alone restored normal NC101 growth kinetics in MM and significantly enhanced growth at 24 h (Figure 1C). NC101 grew when subcultured from MM to LB, indicating NC101 does not have a global growth defect (Figures 1A–C). Together, these data suggest that NC101 is an NA auxotroph.

### Nicotinic Acid Auxotrophy Is Not a Defining Feature of Non-toxigenic *E. coli*

**FIGURE 2:**
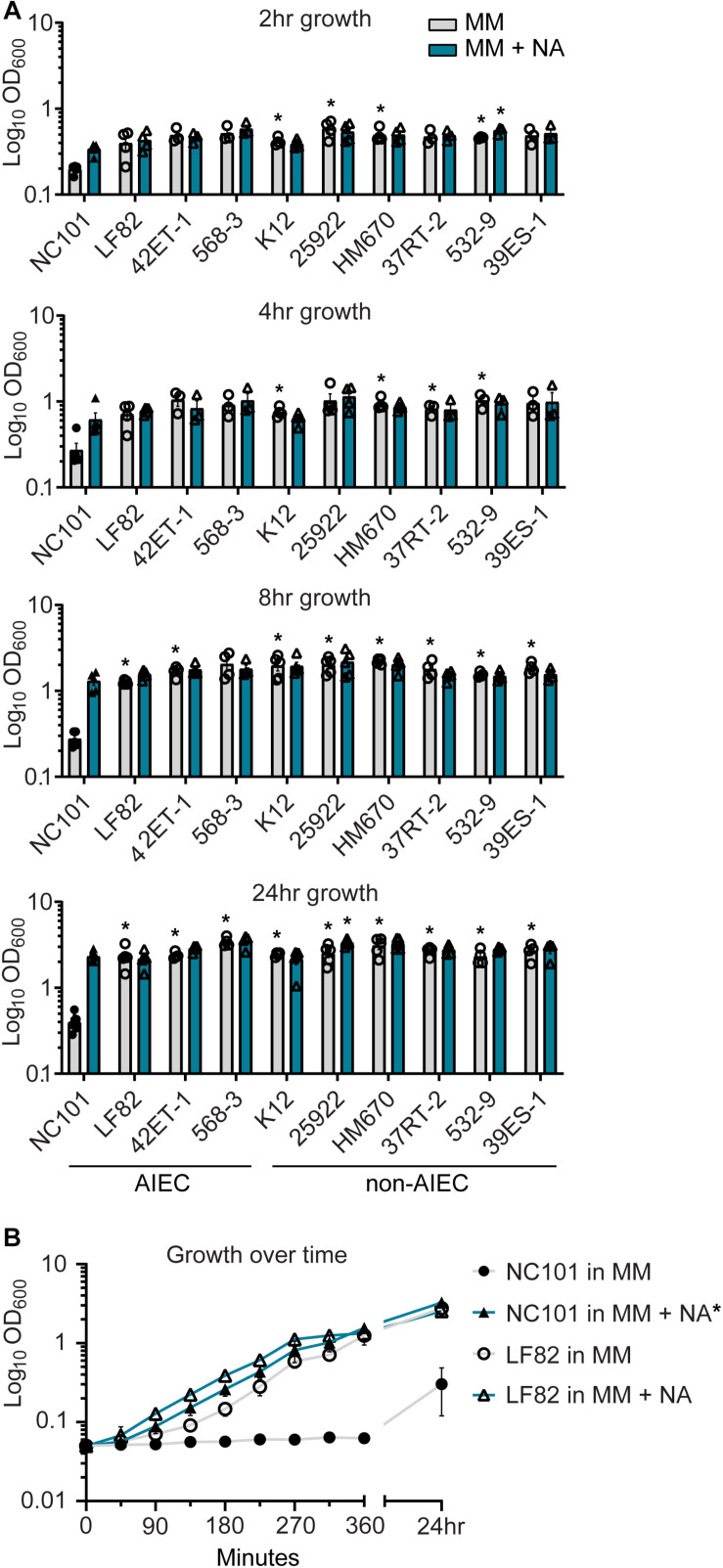
Nicotinic acid (NA) auxotrophy is not common among non-toxigenic *Escherichia coli* strains, including prototypic adherent-invasive *E. coli* (AIEC) LF82. **(A)** AIEC and non-AIEC *E. coli* isolates were grown in minimal media (MM) with and without NA. Growth was evaluated at 2, 4, 8, and 24 h by OD_600_. **(B)** Growth curve of wild-type NC101 and LF82 (human-derived AIEC) in MM with and without NA. **(A)** Bars (*n* = 3–6) or **(B)** points (*n* = 3) depict mean ± SEM. **(A)** Strains grown in MM were compared to NC101 grown in MM, and strains grown in MM + NA were compared to NC101 grown in MM + NA. Significance (*) is shown compared at **(A)** each time point or **(B)** 24 h and was determined at *p <* 0.05 using a one-way ANOVA with Dunnett’s T3 multiple comparisons test.

Resident non-toxigenic *E. coli* are common among the intestinal microbiota, and many are considered commensal strains (Tenaillon et al., 2010; Mirsepasi-Lauridsen et al., 2019). Yet, other *E. coli* (e.g., AIEC) are associated with chronic intestinal inflammation and may be referred to as pathobionts (Martinez-Medina and Garcia-Gil, 2014; Mirsepasi-Lauridsen et al., 2019). We questioned whether NA auxotrophy was shared across clinically derived non-toxigenic *E. coli*. In addition to evaluating model *E. coli* strains (K12 and 25922), we evaluated clinical specimens isolated from the intestinal mucosa of IBD or non-IBD patients (*E. coli* LF82, 42ET-1, 568-3, HM670, 37RT-2, 532-9, and 39ES-1) (Darfeuille-Michaud et al., 1998; Martin et al., 2004; Baumgart et al., 2007) (Table 1). These clinical isolates have been characterized in the lab from which they originated for AIEC status, and at least partial genome sequences are available for all strains (Baumgart et al., 2007). To determine the extent of NA dependency among these strains, we passaged isolates in MM with and without NA and assessed growth by measuring optical density (OD_600_) at 2, 4, 8, and 24 h (Figure 2A). We again observed that NC101 had a growth defect in MM, detectable at 2 h, and continuing through 24 h (Figure 2A). However, all examined laboratory strains and clinical isolates grew in MM with and without NA (Figure 2A). Therefore, NA auxotrophy does not appear to be a defining characteristic shared by non-toxigenic resident intestinal *E. coli*.

LF82 is a well-known human-derived AIEC strain that can grow in MM without NA (Darfeuille-Michaud et al., 1998) (Figures 2A,B). We directly compared the growth kinetics of NC101 and LF82 in MM with and without NA (Figure 2B). While early growth of LF82 in MM was minimally enhanced by NA, this difference was indistinguishable by 6 h (Figure 2B). Thus, the prototypic AIEC strain LF82 does not exhibit NA auxotrophy. Combined with our findings in Figure 2A, we conclude that NA auxotrophy is not an AIEC-defining feature.

### NC101 Has a Defect in the *de novo* NAD Biosynthesis Pathway

**FIGURE 3:**
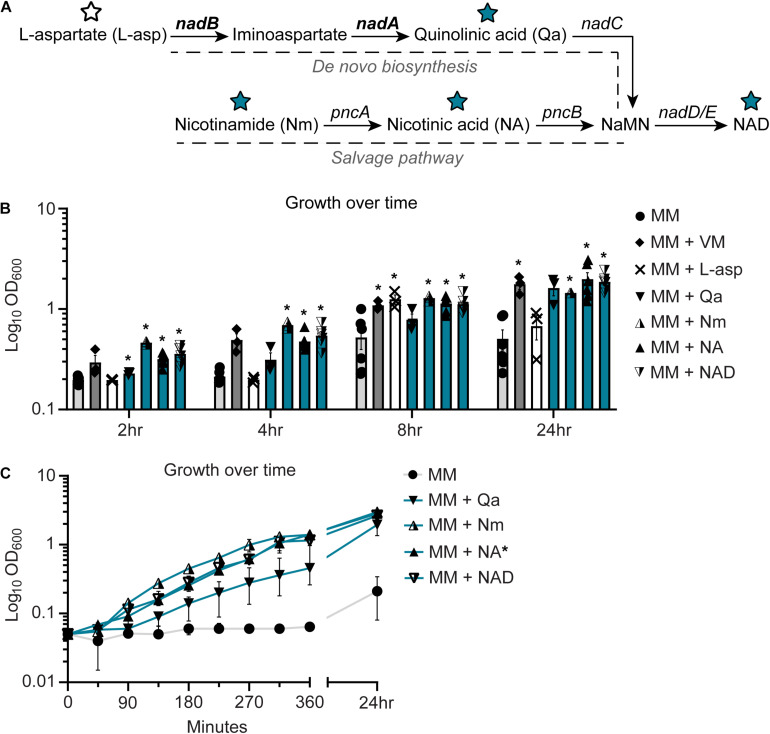
NC101 has a defect in the *de novo* nicotinamide adenine dinucleotide (NAD) biosynthesis pathway. **(A)** Illustration of NAD biosynthesis pathway in *Escherichia coli*, including pathway intermediates and genes involved. Intermediates tested in *E. coli* growth assays are indicated by stars (blue restored growth, white did not). Bolded genes, *nadA* and *nadB*, are predicted to be responsible for NC101 auxotrophy. **(B)** Wild-type NC101 was grown in minimal media (MM), MM + vitamin mix (VM), or MM + NAD biosynthesis pathway intermediates: L-aspartate (L-Asp), quinolinic acid (Qa), nicotinamide (Nm), nicotinic acid (NA), and NAD. Culture density was evaluated at 2, 4, 8, or 24 h by OD_600_. **(C)** Growth curve of NC101 in MM with or without Qa, Nm, NA, or NAD. **(B)** Bars (*n* = 3–6) or **(C)** points (*n* = 3) depict mean ± SEM. Significance (*) is shown compared to NC101 growth in MM at **(B)** each time point or **(C)** 24 h and was determined at *p <* 0.05 using a one-way ANOVA with Dunnett’s T3 multiple comparisons test.

Nicotinic acid is a precursor for NAD biosynthesis (Bouvet et al., 2017). NAD is an electron carrier and an essential cofactor for bacterial metabolism (Bouvet et al., 2017). In *E. coli* and related bacteria, NAD can be synthesized *de novo* from L-aspartate (L-asp) through the generation of quinolinic acid (quinolinate, Qa). In this process, quinolinate synthases A and B (encoded by *nadA* and *nadB*, respectively) catalyze the oxidation of L-asp to iminoaspartate and condensation with dihydroxyacetone phosphate to generate quinolinate (Bouvet et al., 2017). Quinolinate is converted to nicotinic mononucleotide (NaMN) by a *nadC* encoded enzyme and ultimately NAD via enzymes encoded by *nadD* and *nadE* (Bouvet et al., 2017). NAD biosynthesis can also occur through salvage pathways that utilize vitamin precursors like NA or nicotinamide (Nm) (Liu et al., 1982; Di Martino et al., 2013; Bouvet et al., 2017) (Figure 3A).

Since, NA restored the growth of NC101, we predicted that NC101 had a defect within the NAD biosynthesis pathway. To determine whether this was the case, we assessed NC101 growth in MM supplemented with key NAD biosynthesis intermediates: L-asp, Qa, Nm, NA, and NAD. L-asp failed to consistently sustain NC101 growth in MM. Conversely, Qa sustained NC101 growth in MM, and Nm, NA, and NAD significantly restored growth across all time points (Figure 3B). Growth curves revealed the kinetics of enhanced NC101 growth in the presence of the restorative NAD biosynthesis intermediates: Qa, Nm, Na, and NAD (Figure 3C). When examining the NAD biosynthesis pathway, this indicated that NC101 likely had a defect in the NAD biosynthesis genes *nadA* or *nadB* (Figure 3A).

### Nicotinic Acid Auxotrophy in NC101 Is Linked to a Mutation in NAD Biosynthesis Gene *nadA*

**FIGURE 4:**
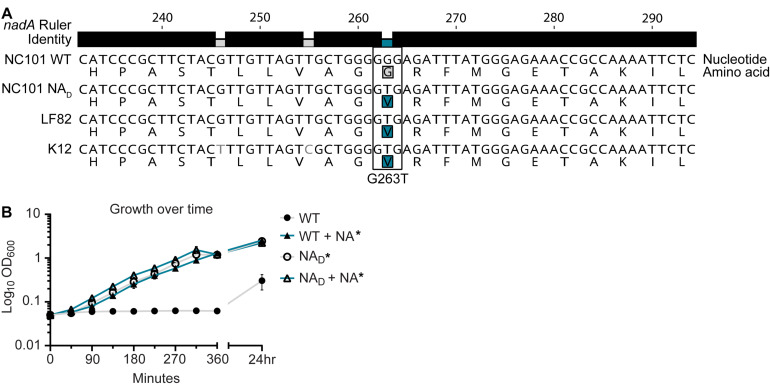
A missense mutation in nicotinamide adenine dinucleotide (NAD) biosynthesis gene *nadA* confers nicotinic acid (NA) auxotrophy in NC101. **(A)** Genetic alignment of partial *nadA* sequence from NA auxotrophic [Wild-type (WT) NC101] and prototrophic (NC101 NA_*D*_, LF82, and K12) *E. coli*. Nucleotide and amino acid sequences, noted by one-letter abbreviations, are shown. The ruler displays nucleotide position of coding sequence. The identity bar displays regions of similarity (black) or dissimilarity (gray or blue). The highlighted amino acids show the region of noted dissimilarity (*nadA* G263T) between NA auxotrophic (gray) and prototrophic (blue) *E. coli*. **(B)** A growth curve of WT NC101 and prototrophic revertant NC101 strain (NA_*D*_) in minimal media (MM) with and without NA. Growth was measured by culture optical density, OD_600_. Points depict mean ± SEM (*n* = 4). Significance (*) is shown compared to NC101 growth in MM at 24 h and was determined at *p* < 0.05 using a one-way ANOVA with Dunnett’s T3 multiple comparisons test.

After our growth supplementation assays revealed a likely defect in *nadA* or *nadB*, we sought to identify the genetic factor(s) responsible for NA auxotrophy in NC101. We performed whole-genome sequencing on WT NC101 and compared the sequence to prototrophic *E. coli*, LF82 and K12. Sequencing revealed that WT NC101 has a missense mutation in *nadA* (T263G) that was associated with auxotrophy (Figure 4A).

To further validate the genetic determinants of NC101 NA auxotrophy, we generated a prototrophic strain by passaging WT NC101 on MM agar plates in the absence of NA (Li et al., 2012). Sequencing of a selected spontaneous prototrophic revertant, termed NA_*Derivative*_ or NA_*D*_ NC101, revealed that NA_*D*_ NC101 had a single nucleotide substitution in *nadA* (G263T, compared to WT) that matched the prototrophic *E. coli* strains LF82 and K12 (Figure 4A). It is important to note that NA_*D*_ NC101 also had a silent mutation in an intergenic region that was absent from WT NC101, but we predict this mutation had no impact on NA_*D*_ NC101 prototrophy (Accession #CP072786) (Table 3). To support that NA auxotrophy is due to the observed *nadA* mutation, our whole-genome sequencing revealed that two other NC101 spontaneous prototrophic revertants had missense mutations in *nadA* – one of which shared the same *nadA* (G263T, compared to WT) nucleotide substitution as NA_*D*_ NC101 (accession #CP072785 and #CP072784) (Table 3).

To confirm that the NA_*D*_ revertant restored prototrophy, we grew WT NC101 and NA_*D*_ in MM with and without NA. NA_*D*_ grew significantly better in MM vs. WT NC101 at 24 h (Figure 4B). Growth of NA_*D*_ in the absence of NA was the equivalent to WT grown with added NA, as noted by overlapping growth curves (Figure 4B). The addition of NA did not significantly enhance NA_*D*_ growth in MM, suggesting NA auxotrophy was successfully eliminated in this strain (Figure 4B). These findings are consistent with the literature, which indicates NadA is important for NAD biosynthesis, and mutations in *nadA* can drive NA auxotrophy in *E. coli*, *Shigella* spp., and *Salmonella* spp. (Bergthorsson and Roth, 2005; Prunier et al., 2007b; Di Martino et al., 2013; Bouvet et al., 2017). Therefore, our data support that NC101 NA auxotrophy is due to a mutation in *nadA*.

### Correcting NA Auxotrophy in NC101 Has Negligible Impact on Bacterial Motility or AIEC-Associated Survival in Macrophages

**FIGURE 5:**
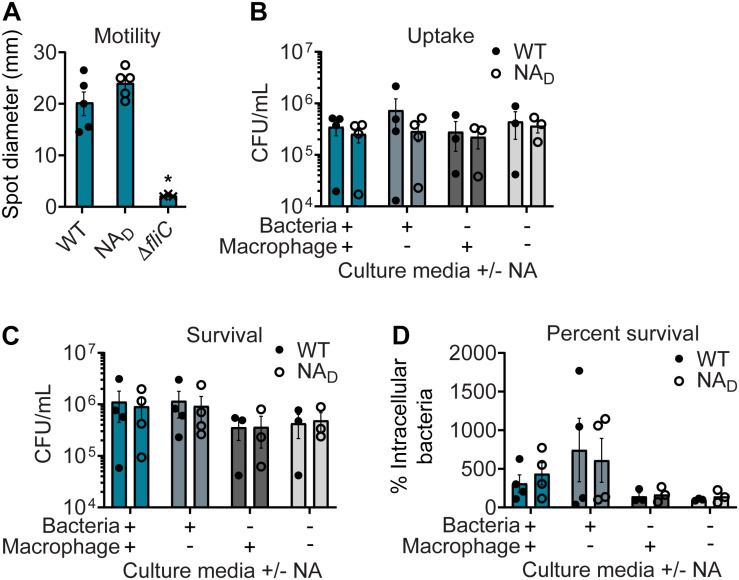
Correcting nicotinic acid (NA) auxotrophy in NC101 has minimal impact on *in vitro* adherent-invasive *Escherichia coli* (AIEC) function. **(A)** Wild-type (WT) NC101, prototrophic NC101 NA_*D*_, and non-motile control NC101 Δ*fliC* were grown on minimal media (MM) soft agar plates with NA. Diameters of motility swarm spots (mm) were measured at 8 h (*n* = 3–5). **(B–D)** J774A.1 murine macrophages were infected at a multiplicity of infection (MOI) = 10 with WT or NA_*D*_. Bacterial culture media before infection (“Bacteria”) or cell culture media during infection (“Macrophage”) were with or without NA supplementation. Numbers of bacteria are shown at **(B)** 1 h or **(C)** 24 h post-infection as Log_10_ colony-forming units (CFUs)/ml. **(D)** Percent survival = [(CFU/ml at 24 h)/(CFU/ml at 1 h] × 100 (*n* = 3–4). Bars depict mean ± SEM. Significance (*) is shown compared to WT NC101 and was determined at *p <* 0.05 using a **(A)** one-way ANOVA with Dunnett’s T3 multiple comparisons test or **(B–D)** Welch’s *t*-test.

To determine if correcting NA auxotrophy impacted AIEC physiology and interactions with mammalian cells, we assessed WT and NA_*D*_ NC101 for motility and survival in macrophages. Motility is not an AIEC-defining feature, but hypermotility has recently been linked to changes in AIEC:host interactions (Martinez-Medina and Garcia-Gil, 2014; Elhenawy et al., 2019). Due to the WT NA auxotrophy, motility was only assessed on MM agar plates supplemented with NA. There was no significant difference in motility between WT and NA_*D*_ NC101 in the presence of NA (Figure 5A). However, the motility of both WT and NA_*D*_ NC101 differed significantly from the non-motile control mutant, NC101 *ΔfliC* (flagellar filament structural protein) (Elhenawy et al., 2019) (Figure 5A).

A key feature of AIEC is enhanced survival in macrophages, a characteristic linked to their pro-inflammatory activities (Darfeuille-Michaud et al., 2004; Kittana et al., 2019). To evaluate whether the identified *nadA* mutation impacted AIEC-defining survival in macrophages, WT and NA_*D*_ NC101 were subcultured in MM with and without NA and used to infect macrophage cell cultures, which were also maintained in the presence or absence of NA. Despite the expected differences in culture densities between WT and NA_*D*_ strains upon subculturing in MM without NA (Figure 1C), we could obtain a sufficient amount of WT NC101 to infect with an equivalent MOI for all experiments. Baseline macrophage cell culture media contains an excess of NA, so as expected, there were not WT NC101 survival defects in the infection assay. Importantly, we found there were no significant differences in AIEC intramacrophage uptake (1 h) or survival (24 h) between WT and NA_*D*_ NC101 in the presence or absence of NA supplementation (Figures 5B–D). Therefore, eliminating NA auxotrophy in NA_*D*_ NC101 had negligible impact on these AIEC-associated functions.

## Discussion

Inflammatory bowel disease pathogenesis differs between patients in terms of the distribution of intestinal inflammation and clinical severity (Khor et al., 2011). The intestinal microbiota influences IBD development (Sartor, 2008; Dogan et al., 2014; Baümler and Sperandio, 2016; Hughes et al., 2017; Caruso et al., 2020). So, IBD heterogeneity could be driven by microbial strain level differences that alter the functional capacity of the intestinal microbiota (Khor et al., 2011). Common members of the mammalian microbiota, like *E. coli*, are associated with human intestinal disease (Tenaillon et al., 2010; Mirsepasi-Lauridsen et al., 2019). Analysis of metabolic features largely distinguishes commensal intestinal *E. coli* strains from those known to induce intestinal and extraintestinal disease (Monk et al., 2013). However, less is known about the distinguishing features of pathobionts, which only cause disease under specific circumstances (e.g., during chronic host inflammation) (Martinez-Medina and Garcia-Gil, 2014; Mirsepasi-Lauridsen et al., 2019).

AIEC are pathobionts that exacerbate intestinal disease (Martinez-Medina and Garcia-Gil, 2014; Mirsepasi-Lauridsen et al., 2019). The evolutionary history of AIEC differs from commensal *E. coli* strains, which is reflected by the pro-inflammatory nature of AIEC (Martinez-Medina and Garcia-Gil, 2014; Bouvet et al., 2017; Elhenawy et al., 2019; Mirsepasi-Lauridsen et al., 2019). There is some phylogenetic linkage between AIEC and extraintestinal pathogenic *E. coli* (Nash et al., 2010). However, despite their ability to induce disease, AIEC do not harbor traditional virulence factors commonly found in pathogenic *E. coli* (Baumgart et al., 2007). Furthermore, due to AIEC genetic diversity, the genetic determinants for their pro-inflammatory functions in IBD are mostly unknown (Nash et al., 2010; Dogan et al., 2014; Martinez-Medina and Garcia-Gil, 2014). So, strain-specific studies on AIEC function are imperative to understanding their role in disease development.

Herein, we illustrate that the AIEC strain NC101 (phylotype B2) is an NA auxotroph due to a missense mutation in NAD biosynthesis gene *nadA*. These findings are significant, as NC101 is an established AIEC often used for studies on IBD and CRC; yet, NA auxotrophy in NC101 has not been defined (Arthur et al., 2012; Tchaptchet et al., 2013; Dogan et al., 2014; Dejea et al., 2018; Ellermann et al., 2019; Gogokhia et al., 2019; Schmitz et al., 2019). The underlying advantage of the *nadA* mutation in NC101 is unclear, as many *E. coli* isolates are prototrophic (Figure 2; Bouvet et al., 2017). However, NA auxotrophy was found to be common among urinary tract *E. coli* isolates and the B2 phylotype of *E. coli* strains that are usual intestinal inhabitants (Domergue et al., 2005; Bouvet et al., 2017). These data suggest NA auxotrophy likely confers a strain-specific selective advantage in the host.

Due to their metabolic and phenotypic diversity, *E. coli* strains remarkably adapt to an array of environments (Bouvet et al., 2017). Microenvironmental pressures influence pathoadaptive mutations in a niche-specific manner (Di Martino et al., 2013; Elhenawy et al., 2019). Genome reduction or loss-of-function mutations may facilitate adaptation to the intestinal microenvironment, as the decrease in biosynthetic cost of compounds likely provides an advantage when key nutrients are consistently present within an environment (Moran, 2002; Nayfach et al., 2019). For example, in *E. coli*, the salvage pathway for NAD biosynthesis takes precedence over *de novo* NAD biosynthesis when precursors (i.e., NA and Nm) are readily available (Bouvet et al., 2017). So, it is possible that an abundance of NA in the murine diet permitted loss of NadA function and improved NC101 fitness. It is also possible that among the many stochastic mutations experienced by *E. coli* strains, this *nadA* mutation simply conferred no benefit or detriment, allowing NC101 to persist as a resident microbe of the murine gastrointestinal tract.

Besides reducing biosynthetic cost, NA/NAD play a key role in virulence and signaling across various microbial species, including *E. coli*, *Shigella* spp., *Candida glabrata*, *Bordetella pertussis*, and *Legionella pneumophila* (Domergue et al., 2005; Prunier et al., 2007a,b; Cannizzo et al., 2011; Li et al., 2012; Di Martino et al., 2013; Edwards et al., 2013; Bouvet et al., 2017). NA auxotrophy in these species is mostly due to mutations in *nadA* and *nadB*, which suggests that convergent evolution could have given rise to an NA requirement across microbes (Domergue et al., 2005; Prunier et al., 2007a,b; Cannizzo et al., 2011; Li et al., 2012; Di Martino et al., 2013; Edwards et al., 2013; Bouvet et al., 2017; Seif et al., 2020). However, the functional consequences of NA auxotrophy promoting mutations appear to be uniquely important to each microbial species and niche (Seif et al., 2020).

In *Shigella* spp., a pathogen but close relative of nonpathogenic *E. coli*, NAD biosynthesis gene products inhibit the secretion of type 3 secretion system (T3SS) effectors and decrease intracellular invasion (Prunier et al., 2007a,b; Di Martino et al., 2013). Thus, loss of functional NAD biosynthesis genes provides a virulence advantage in *Shigella* spp. (Prunier et al., 2007a,b). Conversely, our results demonstrate that NA auxotrophy does not impact NC101 survival in macrophages (a pro-inflammatory and key defining feature of AIEC) (Figure 5). Like many AIEC, NC101 lacks a T3SS (accession #CP072787). Thus, unlike *Shigella* spp., NC101 cellular invasion and intramacrophage survival are independent of T3SS effector delivery. However, NA auxotrophy may influence AIEC function in other ways.

As seen in some species, auxotrophy can improve cellular metabolic homeostasis, protect against intracellular accumulation of reactive oxygen species, regulate signal transduction, influence colonization, and impact microbial community stability (Utsumi et al., 1994; Edwards et al., 2013; Bouvet et al., 2017; Seif et al., 2020). In *E. coli*, NA can regulate the EvgA/EvgS two-component regulatory system that drives multidrug resistance and acid tolerance (Utsumi et al., 1994; Edwards et al., 2013). While it was shown in urinary tract infection models that NAD auxotrophy does not impact *E. coli* colonization, NA auxotrophic strains have been shown to outcompete prototrophic strains in coculture competition assays (Bouvet et al., 2017). Thus, the advantage of NC101 NA auxotrophy is likely context dependent and should be the subject of future studies. Interestingly, *nadA/B* were listed as potential virulence factors in an IBD patient-derived AIEC (Nash et al., 2010). So, while NA auxotrophy is not ubiquitous across all AIEC and *E. coli* isolates, NA auxotrophy may influence the functional features of certain AIEC. Therefore, correcting NA auxotrophy in other host-adapted *E. coli* could globally enhance functional studies and provide further understanding of pathobiont-specific features.

The intestinal microbiota comprises a large/diverse collection of host-associated microbes, microbial genes, and products (Lopez et al., 2021). Our lab and others have been interested in pro-inflammatory and pro-carcinogenic molecules derived from intestinal bacteria, namely, yersiniabactin and colibactin (Arthur et al., 2012; Ellermann et al., 2019). Many host-influencing microbia-derived molecules, often termed specialized metabolites, are produced by sophisticated multi-enzymatic machinery encoded by bacterial biosynthetic gene clusters. By nature, many of these specialized metabolites are difficult to isolate and purify in sufficient amounts for functional analysis. However, their production can often be activated by nutrient deficiency (Hood and Skaar, 2012; Sharon et al., 2014; Baümler and Sperandio, 2016). Therefore, studies on specialized metabolites and their interactions with host cells often require precise nutrient manipulation to study *in vitro*. To optimize the production of these unique bioactive molecules and reduce nonessential media components that complicate purification, we have identified the MM components necessary to grow the model AIEC NC101 and generated an NC101 strain no longer restricted by NA auxotrophy. This strain, NC101 NA_*D*_, can easily be cultured in MM for functional studies or used to purify AIEC specialized metabolites. Ultimately, this strain, NA_*D*_ NC101, can now serve as a research tool to investigate how precise nutrient manipulation impacts AIEC behavior under MM conditions.

Auxotrophies have been used to identify strains and determine microbial lifestyles (e.g., commensal or pathogenic) (Seif et al., 2020). Thus, defining the nutritional needs of host-adapted microbes has been imperative for functional studies on the microbiota. However, the identification of *in vitro* nutrient requirements is often strain-specific and can be experimentally challenging, which complicates the identification of genetic determinants underlying auxotrophy (Seif et al., 2020).

Our work highlights the genetic basis for NA auxotrophy in AIEC NC101. Correcting NC101 NA auxotrophy will (1) enable precise nutrient manipulation for *in vitro* studies on AIEC as they relate to IBD and CRC, (2) inform culture-based methods to evaluate the function of other auxotrophic gut microbiota members and their metabolites, and (3) facilitate small-molecule isolation and purification from the pro-inflammatory and pro-carcinogenic strain NC101. Intriguingly, our findings pose outstanding questions key to understanding the selective advantage of auxotrophy in microbiota members. Long-term, we expect our findings will contribute to the identification of microbiota-derived prognostic and therapeutic targets for human digestive diseases.

## Data Availability Statement

The datasets presented in this study can be found in online repositories. The names of the repository/repositories and accession number(s) can be found in the article/Supplementary Material.

## Author Contributions

LL, CJB, CAB, and JA contributed to the conceptualization and design of the study. LL was responsible for the data curation, formal analysis, visualization, and generation of the first draft. CJB, CAB, and JW wrote the sub-sections of the methods and materials. LL and JA were involved in the supervision and validation of the study. All authors involved in the investigation, reviewed, revised, and approved the submitted manuscript.

## Conflict of Interest

The authors declare that the research was conducted in the absence of any commercial or financial relationships that could be construed as a potential conflict of interest.

## Footnotes

1 https://github.com/nanoporetech/medaka
