# Supplementary material for: A nadA Mutation Confers Nicotinic Acid Auxotrophy in Pro-carcinogenic Intestinal Escherichia coli NC101
Source: Front Microbiol. 2021 Jun 2;12:670005. doi: 10.3389/fmicb.2021.670005 (PMC8207962; doi:10.3389/fmicb.2021.670005)

CLC bio

Table of contents

1. Mapping summary report 3
    1.1 Summary statistics 3
    1.2 Distribution of read length 4
    1.3 Distribution of mapped read length 5
    1.4 Distribution of un-mapped read length 6
    1.5 Paired reads distance distribution 7

1. Mapping summary report

1.1 Summary statistics

	Count	Percentage of reads	Average length	Number of bases	Percentage of bases
References	1	-	5,030,087.00	5,030,087	-
Mapped reads	2,665,212	48.68%	127.88	340,818,876	91.55%
Not mapped reads	2,809,296	51.32%	11.20	31,461,870	8.45%
Reads in pairs	2,473,968	45.19%	152.19	312,517,012	83.95%
Broken paired reads	191,244	3.49%	147.99	28,301,864	7.60%
Total reads	5,474,508	100.00%	68.00	372,280,746	100.00%

1.2 Distribution of read length

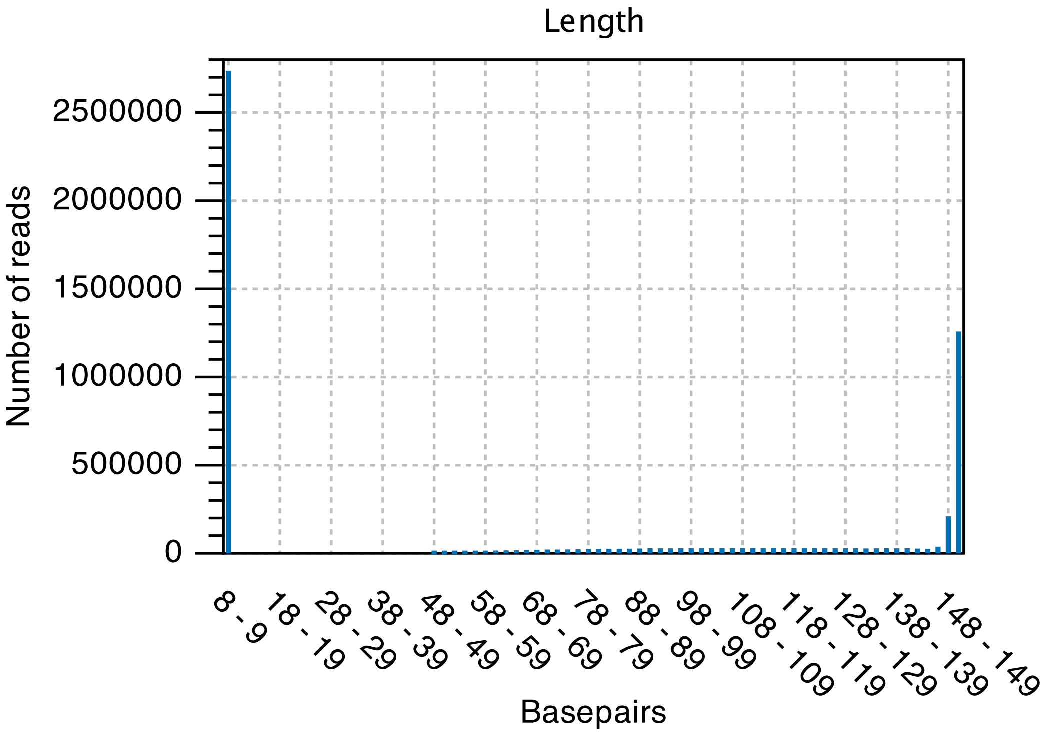

1.3 Distribution of mapped read length

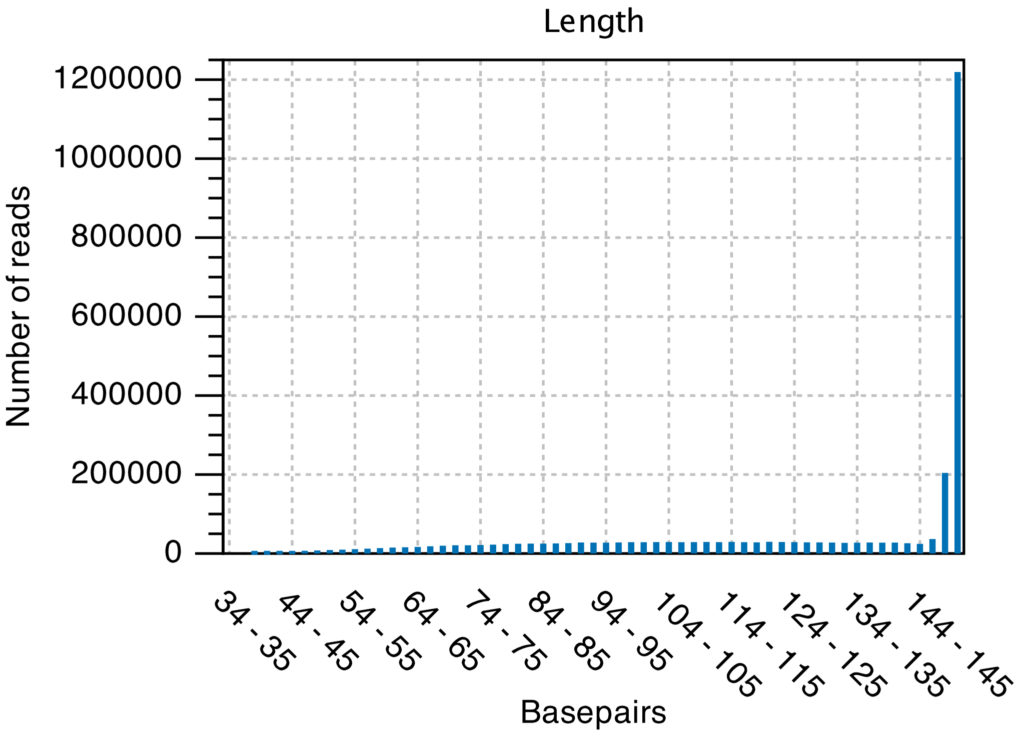

1.4 Distribution of un-mapped read length

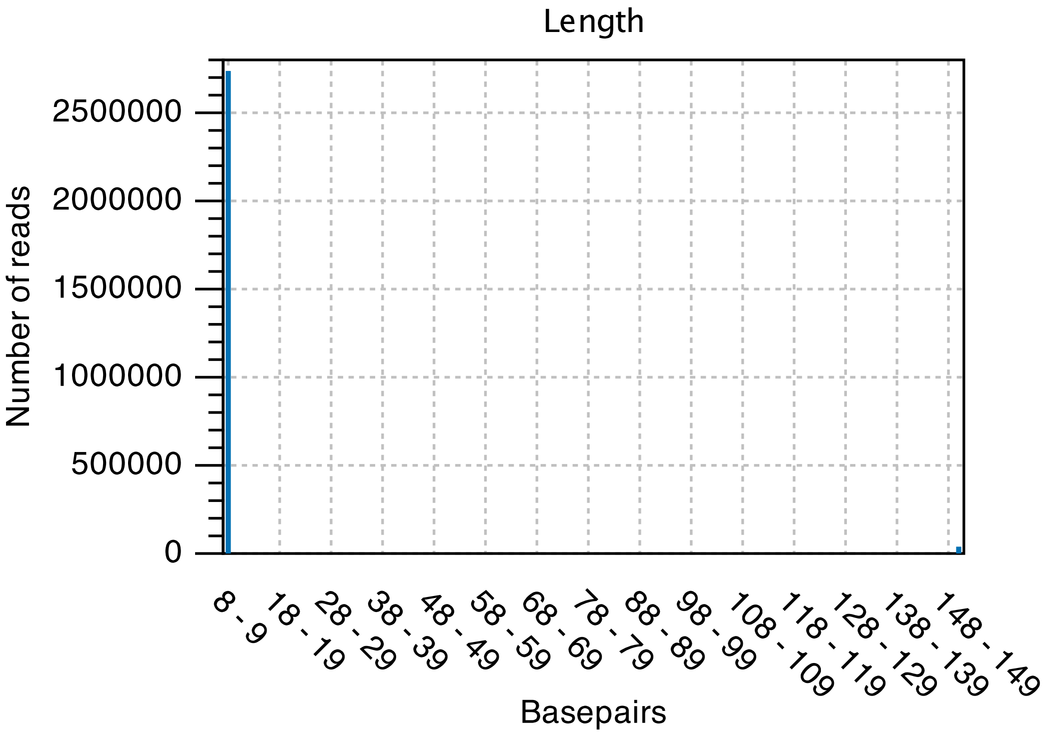

1.5 Paired reads distance distribution

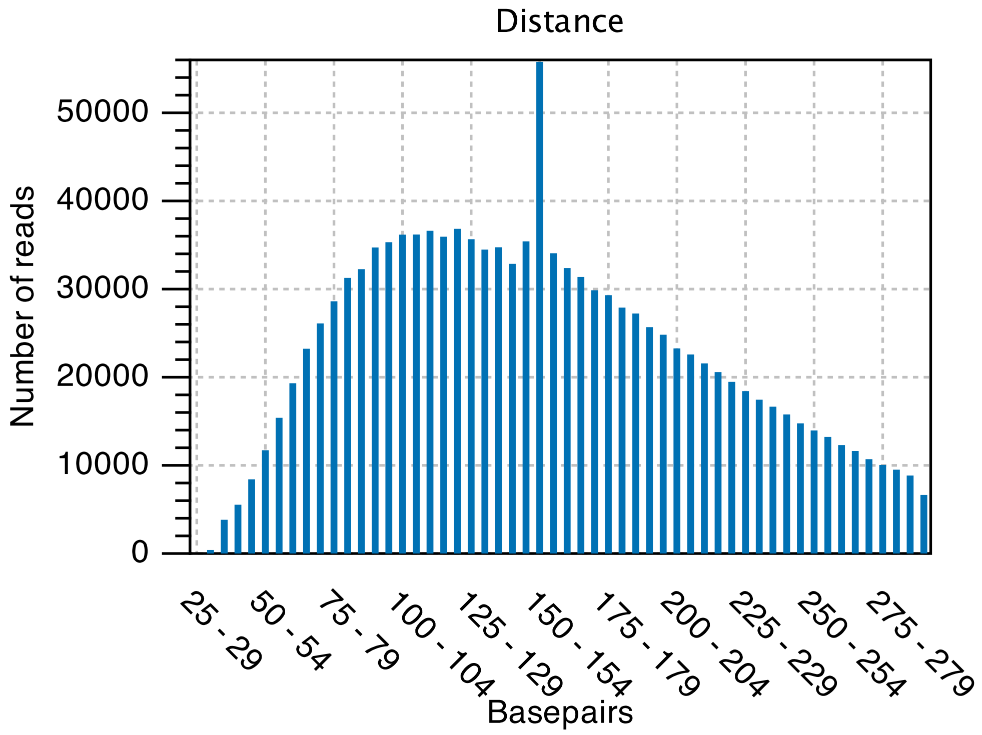

Supplement: Supplementary file 1 [file Data_Sheet_1.PDF]
